# Loss of both wobbleU_34_ modifications in mcm^5^s^2^U tRNAs impairs rRNA biosynthesis, growth, and development in *Arabidopsis thaliana*

**DOI:** 10.3389/fpls.2025.1681927

**Published:** 2025-12-03

**Authors:** Yumi Nakai, Yukio Kurihara, Yuko Makita, Gorou Horiguchi, Kosei Iwabuchi, Akiko Harada, Masato Nakai, Takato Yano

**Affiliations:** 1 Department of Biochemistry, Faculty of Medicine, Osaka Medical and Pharmaceutical University, Osaka, Japan; 2 Synthetic Genomics Research Group, RIKEN Center for Sustainable Resource Science, Yokohama, Japan; 3 Graduate School of Engineering, Maebashi Institute of Technology, Maebashi, Japan; 4 Department of Life Science, College of Science, Rikkyo University, Tokyo, Japan; 5 Research Center for Life Science, College of Science, Rikkyo University, Tokyo, Japan; 6 Department of Biology, Faculty of Medicine, Osaka Medical and Pharmaceutical University, Osaka, Japan; 7 Institute for Protein Research, Osaka University, Osaka, Japan

**Keywords:** tRNA, wobbleU_34_ modification, rRNA biosynthesis, ribosome pausing, *Arabidopsis thaliana*

## Abstract

In eukaryotic cells, the first anticodon uridine base of cytosolic tRNALys(UUU), tRNAGlu(UUC), and tRNAGln(UUG) (wobbleU_34_) is post transcriptionally modified through adding a methoxycarbonylmethyl group and substituting a sulfur at the fifth and second carbons, respectively, to form 5-methoxycarbonylmethyl-2-thiouridine. The simultaneous deletion of these two wobbleU_34_ modifications causes lethality in mice and flies. Here, we report that deletion of both wobbleU_34_ modifications results in severe growth retardation and morphological abnormalities in *Arabidopsis thaliana*. The results of Ribo-seq and RNA-seq analyses indicate that the ribosome occupancy of many transcripts is substantially different in the *Arabidopsis* mutant lacking both wobbleU_34_ modifications compared with the wild type. Gene Ontology analysis shows that genes with altered ribosome occupancy are categorized as having RNA-binding properties. Several pre-rRNA processing precursors accumulate in the mutant lacking both wobbleU_34_ modifications. In the mutant, ribosomes likely pause when the cognate [A/G/C]AA codons of tRNALys(UUU), tRNAGlu(UUC), and tRNAGln(UUG) are positioned at the A site during translation of transcripts encoding proteins involved in pre-rRNA processing, such as *DRH1* and *ATRH7*. These findings suggest that deleting both wobble U_34_ modifications impairs rRNA maturation, leading to the accumulation of rRNA precursors adversely affecting growth and morphogenesis in plants.

## 1 Introduction

Posttranscriptional RNA modifications affect a variety of functions in gene transcription and translation processes to maintain cellular homeostasis (Delaunay and Frye, 2019). Many nucleoside modifications occur in various tRNAs, and modifications mostly found in the first anticodon nucleosides of specific tRNAs help maintain translation efficiency (Suzuki, 2021). The uridine at the first anticodon position of tRNALys(UUU), tRNAGlu(UUC), and tRNAGln(UUG) in the eukaryotic cytosol is modified to 5-methoxycarbonylmethyl-2-thiouridine (mcm^5^s^2^U) (the wobbleU_34_ modification), which comprises a methoxycarbonylmethyl group added to the fifth carbon of the base (mcm^5^) via the Elongator complex (Jaciuk et al., 2023; Abbassi et al., 2024) and a sulfur atom on the second carbon of the base, attached by the intracellular sulfur transport relay system (Nakai et al., 2008; Sokołowski et al., 2024).

The wobbleU_34_ modification in the plant *Arabidopsis thaliana* requires the Elongator complex (Mehlgarten et al., 2010) and the methyltransferase AtTRM9 (Leihne et al., 2011) to form the mcm^5^ group, and two *Arabidopsis URM* genes, *URM11* and *URM12*, are required for the sulfur modification (Nakai et al., 2012, 2019). We previously found that the first pair of true leaves in the *urm11–1 urm12–1* double mutant (referred to as *urm* in this article) or in the *elo3–10* Elongator mutant (referred to as *elo* in this article) of *A. thaliana* were slightly delayed in entering the endoreduplication phase of the cell cycle. The arrangement of mesophyll cells was slightly disturbed compared with that of the wild-type plant (WT) (Nakai et al., 2019).

These two distinct modifications of wobbleU_34_ independently contribute to adjusting the stacking rigidity and flexibility of the codon–anticodon pair structure that specifies Lys, Glu, or Gln (Yarian et al., 2000; Murphy et al., 2004; Duechler et al., 2016; Vendeix et al., 2012; Larsen et al., 2015), and lack of one of the two wobbleU_34_ modifications may have a limited impact on plants (Nakai et al., 2019). The wobbleU_34_ modification may affect translation of almost all gene transcripts, but the effect of the complete loss of both wobbleU_34_ modifications on translation and on plant growth and development remains unknown. Here, we created a triple mutant *tri* (*urm11–1 urm12–1 elo3-10*) lacking both wobbleU_34_ modifications, characterized the mutant phenotypes, and analyzed gene expression and translation using RNA-seq and Ribo-seq, respectively.

## 2 Materials and methods

### 2.1 Plant materials, growth condition, and treatments

*Arabidopsis thaliana* Columbia-0 (Col-0) was used as the WT plant. The *urm* (*urm11–1 urm12-1*) and *elo* mutants (*elo3-10*), both of which have a Col-0 background, were described previously (Nakai et al., 2019). The *tri* mutant (*urm11–1 urm12–1 elo3-10*) was obtained by crossing the *urm* and *elo* strains. The plants were initially cultured on half-concentration Murashige and Skoog (MS) agar medium (Murashige and Skoog, 1962) with 2% sucrose. Water-absorbed seeds were dormant in the dark at 4°C for 3 days, and then sown and cultured under illuminated conditions, with the first day of growth under illumination designated as 0 days after stratification (DAS). Seedlings were also grown under white fluorescent light (∼90-110 µmol/m^2^/s) with a 16-h light/8 h dark cycle at 22°C in a growth chamber BIOTRON LPH 200 (NK System). Seedlings were grown in sterile soil or Rockwool, as previously reported (Nakai et al., 2019).

### 2.2 Ribo-seq and RNA-seq

The Ribo-seq and RNA-seq datasets were acquired as previously described (Kurihara et al., 2018). In brief, the cell extracts prepared from the plant sample 11 DAS were treated with DNase I (Thermo Fisher Scientific) and used for total RNA and ribosome footprint preparations. The total RNA was extracted using TRIzol LS reagent (Thermo Fisher Scientific) and a Direct-zol RNA kit (ZYMO RESEARCH), and libraries for RNA-seq were constructed using a TruSeq Stranded Total RNA Library Prep Kit (Illumina). Purified ribosome footprints were used to construct Ribo-seq libraries as previously described (Kurihara et al., 2018). RNA sequence data were obtained from the RNA-seq and Ribo-seq libraries using HiSeq X (Illumina). RNA-seq reads were mapped to the *Arabidopsis* TAIR11 genome using STAR (Dobin et al., 2013) after rRNA/tRNA removal. The Ribo-seq reads were mapped to the TAIR11 genome using TopHat version 2.1.1. The mapping data from WT and mutant plants were merged into a single dataset, and open reading frames (ORFs) were predicted using RiboTaper v1.2 (Calviello et al., 2016) with 27–29-nt ribosome footprint reads. Read counts for each predicted ORF were normalized using DESeq (Trapnell et al., 2012). All samples were analyzed with two biological replicates.

### 2.3 Generating P site plot using Ribo-seq data and analyzing ribosome footprints

The Ribo-seq data records corresponding to 28- and 29-nt, 100% matched ribosome-protected sequences (ribosome footprints) for each of particular transcript were extracted using local BLAST. The extracted 28- or 29-nt sequences were aligned across the transcript to generate a P site plot with a 12- or 13-nt offset, respectively. Their 3-nt periodicities were confirmed throughout their coding sequences from the first ATG codon to the last codon adjacent to the stop codon. The ribosome footprint counts from the WT and the mutants were normalized based on their total Ribo-seq read counts and compared in association with the particular codons positioned at the P, A, or E site, as well as the −6- or +6-nt site relative to the P site. The ribosome footprint counts for the cognate [A/G/C]AA and [A/G/C]AG codons corresponding to the wobbleU_34_-containing tRNAs positioned at these six positions on the translating ribosomes were separately compared between the WT and the *tri*, *urm*, or *elo* mutants. Their differences were plotted. Unrelated [A/G/C]GA and [A/G/C]GG codons were analyzed and plotted as controls.

### 2.4 Comparing gene expression profiles between mutants and WT

We calculated the fold-change log_2_ values between the normalized mRNA read counts of each mutant and those of the WT (mRNA-FClog_2_) as well as the fold-change log_2_ values between the normalized ribosome-occupied RNA read counts of each mutant and those of the WT (ribo-FClog_2_). The translational efficiency (TE) of each transcript was defined as the ribosome occupancy per transcript. The TE value was calculated by dividing the Ribo-seq read counts by the RNA-seq read counts using DESeq (Calviello et al., 2016). The significant differences in the TE between each mutant and the WT (log_2_ fold-change of the TE, TE_FClog_2_) with a threshold-adjusted *p*-value <0.05 were analyzed using the Benjamini–Hochberg (BH) method. The TE_FClog_2_ value was plotted against the log_2_ value of the normalized mRNA read count (mRNA-NormClog_2_).

### 2.5 Gene Ontology enrichment analysis

Gene Ontology (GO) enrichment was analyzed using DAVID (https://davidbioinformatics.nih.gov/home.jsp) and PANTHER (https://pantherdb.org) against the *Arabidopsis* gene annotation using TAIR11. The false discovery rate for GO items was determined using the BH method, and only items with an adjusted *p*-value < 0.05 were selected. The results of the GO enrichment analysis were visualized using the GO plot (https://wencke.github.io).

### 2.6 Extracting total RNA for northern analysis of *A. thaliana*

Total RNA was prepared from whole plants 11 DAS using an ISOSPIN Plant RNA kit (Nippon-Gene). Approximately 10–20 µg of total RNA was used for northern hybridization analysis, performed as previously described (Kojima et al., 2018). Briefly, the RNA samples were separated on a 1%–1.2% denaturing agarose gel, blotted onto the nylon membrane (Hybond-N^+^, Cytiva), and hybridized with digoxigenin (DIG; Roche Diagnostics GmbH)-labeled DNA probes to detect specific rRNA precursor regions. The primers used to amplify the DIG-labeled DNA probes are shown in **Supplementary Table S4**. All samples were analyzed in triplicate.

### 2.7 cRT-PCR and sequencing of plant RNA

cRT-PCR was performed as previously described (Maekawa et al., 2018). Briefly, total RNA was treated with DNase I, and a circularized library was formed using RNA ligase (TaKaRa Bio). First-strand cDNA was synthesized via reverse transcription using a primer bound to the target 25S rRNA region. PCR was performed using different primer sets. The length of the amplified DNA was confirmed by agarose gel electrophoresis, and the sequences encompassing the junction points formed by RNA circularization were determined by DNA cloning with the Mighty Cloning Kit (TaKaRa Bio) followed by DNA sequencing. The primers and probes used are shown in **Supplementary Table S4**. All samples were analyzed in triplicate.

### 2.8 Histological observations

The entire leaf and subepidermal parenchyma cells were observed as previously described (Nakai et al., 2019). The seeds in the silique were visualized using a clearing agent, ClearSeeTM (Fujifilm WAKO) following the manufacturer’s instructions. Plant tissues were observed under a stereomicroscope All-in-One BZ X-700 (KEYENCE) using the related products. The entire leaf area and leaf cell size were measured, and cells were counted using ImageJ. More than 20 mesophyll cells in the lower epidermal layer, from the midrib of the leaf vein at the center of the leaf blade to the leaf margin, were analyzed for each leaf. The transverse section of the leaf and the upper epidermal tissue were observed using scanning electron microscopy (LSM800, Zeiss), and the number of cells were measured as previously described (Nakai et al., 2019).

### 2.9 Germination assay

The WT and the *tri* seeds were grown on agar medium supplemented with or without gibberellin (GA) (GA3, Fujifilm WAKO). After sowing and stratification, seeds were germinated under normal growth conditions. GA was added to the medium before sowing the seeds at final concentrations of 1 or 10 µM.

## 3 Results

### 3.1 Lack of both wobbleU_34_ modifications in *Arabidopsis* causes severe growth retardation and various morphological changes

**Figure 1:**
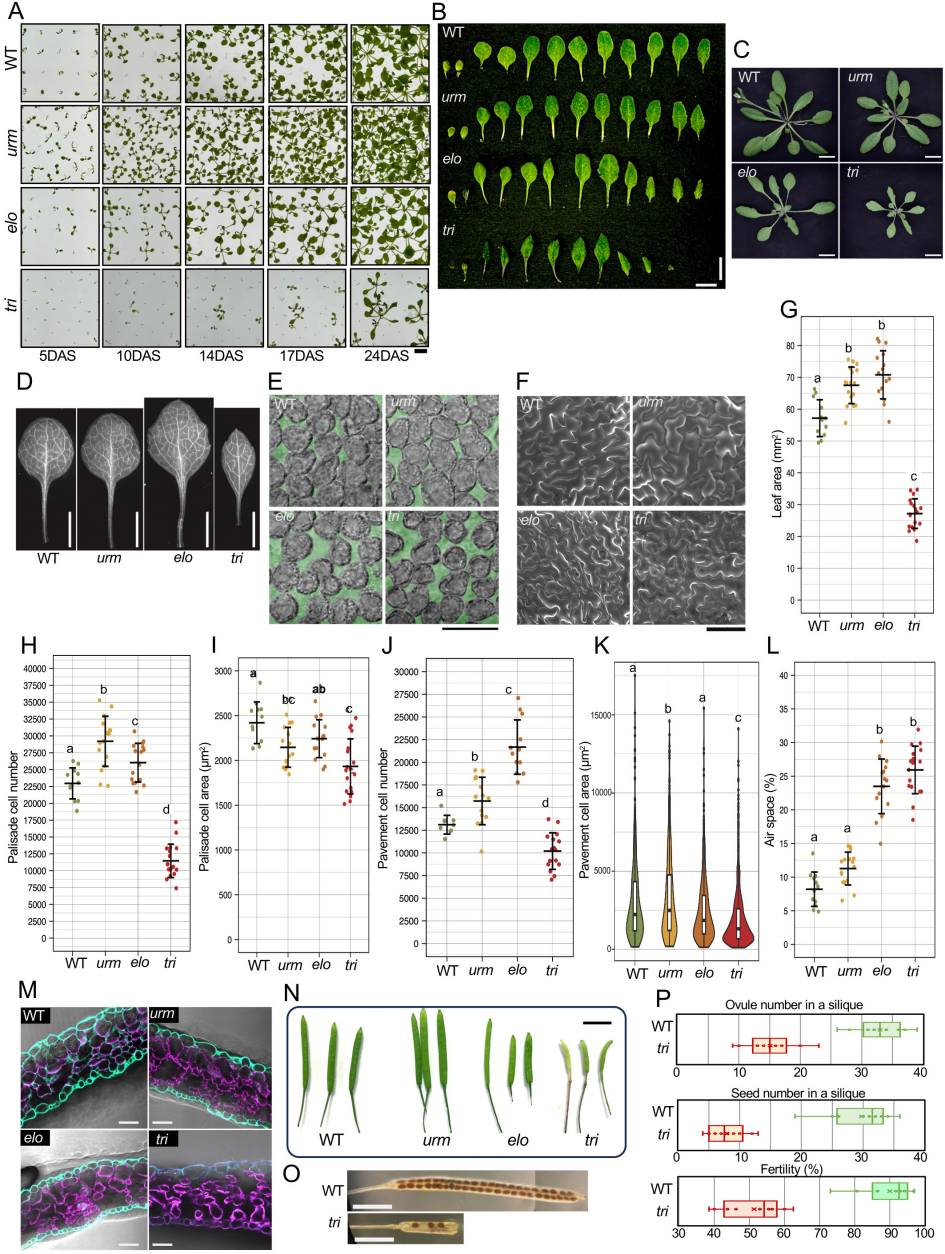
Growth and morphological phenotypes of *Arabidopsis* woblbeU_34_ mutants. **(A)** Growth of WT, *urm*, *elo*, and *tri* plants was compared at 5, 10, 14, 17, and 24 DAS. Scale bar, 1 cm. **(B)** Cotyledons and true leaves of WT and mutants of 21 DAS were aligned. Scale bar, 1 cm. **(C)** Shoots of WT and the wobbleU_34_ modification mutants grown for 26 days. Scale bar, 1 cm. **(D)** First leaves of indicated genotypes. Scale bar, 5 mm. **(E)** Palisade cells in the adaxial subepidermal layer. Air space is indicated in green. Scale bar, 100 μm. **(F)** Adaxial epidermal tissues observed by a scanning electron microscope. Scale bar, 100 μm. **(G–L)** show the quantitative characteristics of the leaf phenotype of the wild type and the wobbleU_34_ modification mutant strains: leaf blade area **(G)**, palisade cell number **(H)**, palisade cell area **(I)**, pavement cell number **(J)**, pavement cell area **(K)**, and proportion of air space **(L)** in the first leaves of indicated genotypes. Numbers of palisade cells in adaxial subepidermal layer **(H)** and of adaxial pavement cells **(J)** were estimated by multiplying the leaf blade area by the corresponding cell density in each leaf. The number of leaves examined was 12 to 18 in **(G, H, I, L)**, and 10 to16 in **(J, K)**, respectively. The adaxial subepidermal layer was examined to measure projected areas of palisade cells and air space. A total of 20 palisade cells were measured per leaf. The number of pavement cells examined ranged from 379 to 1,093 in **(K)**. Means ± s.d. are shown in **(G, H, I, J, L)**. In **(K)**, data are shown using violin plots and box plots. The 25th and 75th percentiles are indicated by lower and upper box edges, respectively, the median by a thick horizontal bar, and maximum and minimum values by whiskers. Different letters in each graph indicate statistically significant differences among genotypes (*p* < 0.05). *p*-values in **(G, H, I, J, L)** were determined using the Tukey honest significant difference test, and those in **(K)** were determined using the Kruskal–Wallis one-way analysis of variance test followed by Dunn’s test with Bonferroni correction. **(M)** Transverse sections of leaves were observed after staining cell walls with Calcofluor White. Magenta indicates chloroplast autofluorescence. Scale bar, 50 µm. **(N)** Whole sheath shape of WT and mutants. Scale bar, 5 mm. **(O)** Representative example of seed attachment in WT and *tri* dishes with transparent sheaths. **(P)** Number of ovaries per silique (top), number of ripe seeds per silique (middle), and fertility rate per silique (bottom) for WT and *tri*. Fertility is expressed as the number of ripe seeds per total number of ovaries per sheath.

By crossing two distinct wobbleU_34_ modification mutants (*urm* and *elo*), we first obtained a mutant line carrying the heterozygous *ELO3*/*elo3–10* alleles with the homozygous *urm11–1 urm12–1* mutations. Then, from the progenies of this line, we successfully obtained the homozygous triple gene mutant (*urm11–1 urm12–1 elo3-10*) and named it the *tri* mutant (**Supplementary Figure S1A**). The growth of the *tri* mutant, lacking the both wobbleU_34_ modifications, was substantially slower from the early stages of germination than that of the *urm* mutant lacking sulfur modification, the *elo* mutant lacking the mcm^5^ modification, and the WT plant (**Figure 1A**; **Supplementary Figure S1A**). The leaf development of the *tri* mutant was even slower than that of the *elo* mutant, with only eight to nine true leaves 21 DAS (**Figure 1B**), and the whole size of the *tri* mutant plant was smaller than that of the other strains (**Figure 1C**). Segregation analysis using seeds obtained from the abovementioned parental line (*urm11–1 urm12–1 ELO3/elo3-10*) confirmed that such slower growth was only observed for the *urm11–1 urm12–1 elo3–10* homozygous triple mutant progenies but not for the *urm11–1 urm12–1 ELO3/ELO3* or the *urm11–1 urm12–1 ELO3/elo3–10* progenies (**Supplementary Figure S1A**).

We performed microscopic observations of leaves to compare their quantitative characteristics using the first pair of true leaves 21 DAS from WT and the mutants lacking the wobbleU_34_ modifications (**Figures 1D–L**). The leaf area of the *tri* mutant was much smaller than that of the WT, whereas those of the *urm* and the *elo* mutants were slightly larger (**Figures 1D, G**). By observing the palisade cells in the adaxial subepidermal layer (**Figure 1E**), cell densities (cell numbers per unit area) were calculated. Total palisade cell numbers per leaf (**Figure 1H**) were estimated by multiplying the cell density by the total leaf area (**Figure 1G**). As a result, the total palisade cell numbers in the *urm* and *elo* mutants were slightly higher than that of the WT, whereas that in the *tri* mutant was substantially lower than the others (**Figure 1H**). In addition, the individual palisade cell areas were slightly reduced in the *urm* and *elo* mutants but were further reduced in the *tri* mutant (**Figures 1E, I**). The pavement cell density of the *tri* and *elo* mutants appeared to be higher than that of the others (**Figure 1F**) because of their smaller individual pavement cell areas (**Figures 1F, K**). Depending on the differences in leaf areas (**Figures 1D, G**), the total pavement cell number per leaf in the *tri* mutant was substantially smaller than that in the others, whereas that of the *elo* mutant was much larger than that of the WT or the *urm* mutant (**Figure 1J**). As a consequence, in the *tri* mutant, both palisade and pavement cells were smaller and fewer per leaf than in the others. These results are consistent with the macroscopic observation that leaves were smaller in the *tri* mutant (**Figures 1B, D**).

The proportion of air space in the *tri* mutant was much larger than that of the WT or the *urm* mutant, and even larger than that of the *elo* mutant (**Figures 1E, L**). The palisade mesophyll cells in the first layer under the epidermis in the *tri* mutant were not arranged in parallel. The overall cell arrangement was sparse compared with that of the other strains (**Figure 1M**), indicating that the mesophyll cells in the leaf tissue of the *tri* mutant were spatially disordered compared with those of the WT and other mutants. Taken together, these results suggest that the deletion of both wobbleU_34_ modifications affects leaf cell proliferation and differentiation, disrupting the balance between area and number of mesophyll and epidermal cells, altering cellular spatial arrangement, and leading to dwarf leaves.

Abnormal morphogenesis was also seen in other organs in the *tri* mutant. Although the siliques shapes of the *urm* and *elo* strains were similar to those of WT (**Figure 1N**), the *tri* mutant pistils had an abnormal shape characterized by shorter valves and a longer gynophore (**Figure 1N**), which is known as the short-valve trait (Nishimura et al., 2005). The *tri* mutant produced mature seeds; however, it produced considerably fewer seeds per silique than the WT (**Figures 1O, P**). Less than half the ovules per silique and mature seeds were produced by the *tri* mutant compared with the WT (**Figure 1P**), and many gametophytes were stunted in the *tri* mutant. In addition, based on the results of segregation analysis for the progenies derived from the heterozygous *ELO3*/*elo3–10* parental line under the homozygous *urm11–1 urm12–1* background as mentioned above, the *tri* seedlings appeared at below the expected ratio of 0.25, suggesting that the *tri* seeds were apt to lose the ability to germinate (**Supplementary Figure S1B**).

To examine the possibility that the observed slow growth phenotype of the *tri* mutant shown in **Figure 1A** results from delayed germination, we performed germination analysis with and without the addition of the gibberellin (GA) in the culture media (**Supplementary Figure S2A**). In the absence of exogenous GA, the *tri* mutant seeds began to germinate at around 48 h after stratification, whereas the WT seeds began to germinate at approximately 24 h. Increasing GA concentrations enhanced germination rates, and this effect was observed similarly in WT and *tri* seeds. However, the delayed germination of the *tri* mutant seeds was not fully complemented even by the addition of 10 µM GA. Moreover, irrespective of the presence or absence of GA in the medium, the slow growth of the *tri* mutant was equally observed (**Supplementary Figure S2B**). Therefore, the slow growth of the *tri* mutant seedlings did not result solely from delayed germination or a defect in GA biosynthesis during germination.

### 3.2 Lack of both wobbleU_34_ modifications strongly affects ribosome occupancy in many transcripts especially in RNA-binding protein-related genes

RNA-seq and Ribo-seq were performed on 11 DAS *Arabidopsis* seedlings to analyze differentially expressed genes between wobbleU_34_ mutants and WT. We calculated the log_2_-fold-change in mRNA expression levels (mRNA-FClog_2_) for each mutant relative to WT to indicate gene expression levels that differed in the mutants during transcription. The log_2_-fold-change in ribosome occupancy (ribo-FClog_2_) was similarly calculated to detect alterations in gene expression levels during translation in the mutants.

**Figure 2:**
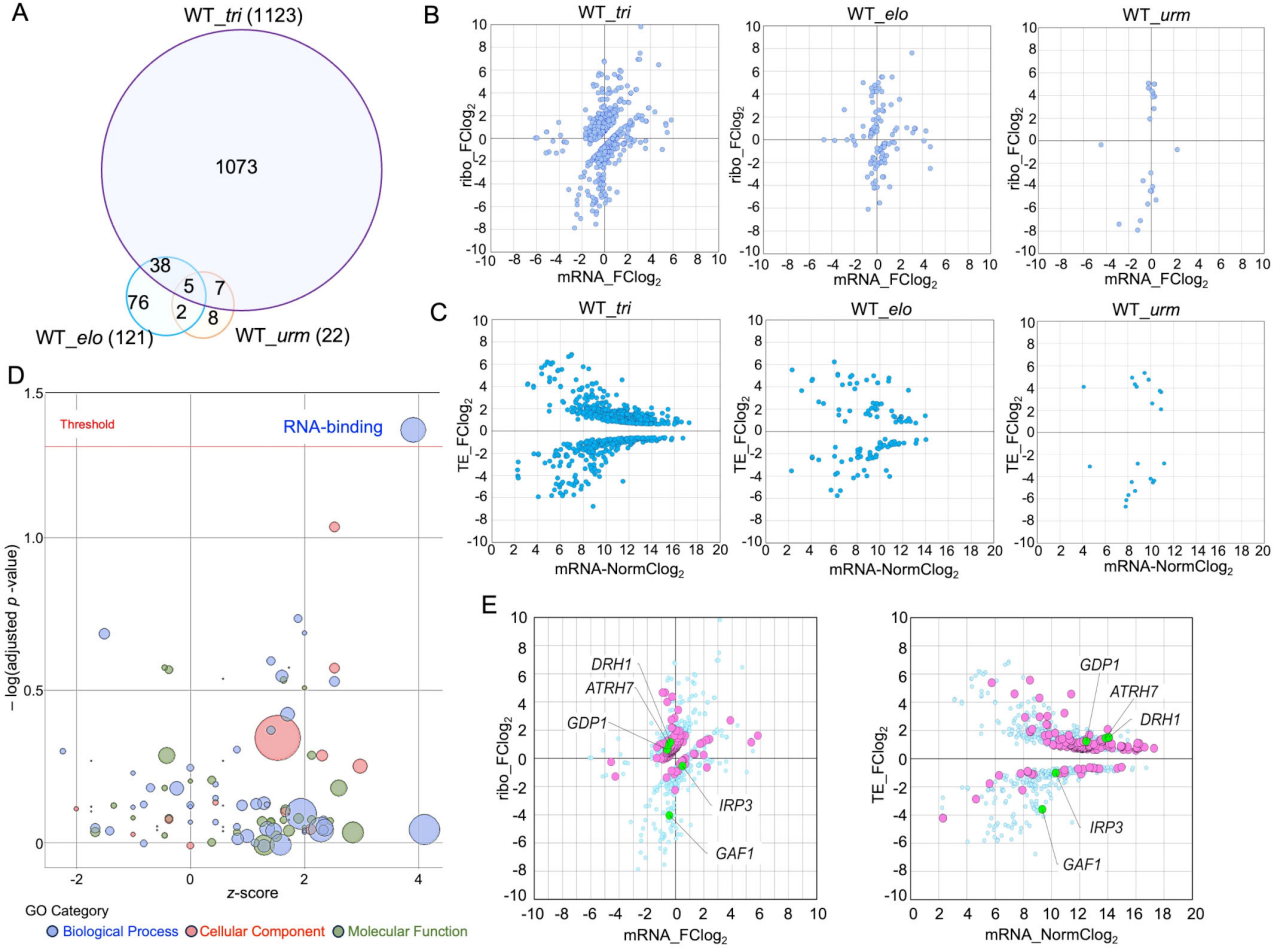
Differential gene expression analysis between the WT and mutants. **(A)** Overlap of transcripts with significant TE_FClog_2_ values between WT and each mutant. Numbers indicate number of significantly differed transcripts in each comparison. **(B)** Log_2_-fold change in mRNA expression (mRNA-FClog_2_) versus log_2_-fold change in ribosome occupancy (ribo_FClog_2_) in each mutant relative to WT. Plots show data for transcripts that exhibited only significant log_2_-fold changes. **(C)** Log_2_-fold change value of change in TE versus normalized log_2_ values of mRNA read counts (mRNA_NormClog_2_) for comparison of variation in expression between strains. **(D)** Gene Ontology analysis of the 1,123 transcripts that significantly differed in *tri*. The X-axis presents the *z*-score, where positive values indicate that GO terms of each category were most likely to be enriched. The y-axis indicates the significance of extracted GO terms as negative log_10_ values of adjusted *p*-values, with higher values indicating greater significance. The threshold was set at an adjusted *p*-value < 0.05. Bubble size indicates number of transcripts, and bubble color indicates type of each GO term: indicated biological process, cellular component, and molecular function, respectively. GO terms are listed in **Supplementary Table S2**. **(E)** Among the 1,123 transcripts (light blue circles), 145 transcripts belonging to the RNA-binding category identified by GO analysis are highlighted by pink and light-green circles in the same plots shown in **(B, C)** (WT_*tri*).

We defined differential ribosome occupancy for each transcript as the TE (Fujita et al., 2019). We calculated the TE by dividing the normalized Ribo-seq read counts by the normalized RNA-seq read counts for each transcript. A total of 1,123 genes were significantly differentially expressed in the *tri* mutant dataset (WT_*tri*), 121 in the *elo* strain (WT_*elo*), and 22 in the *urm* strain (WT_*urm*) compared with the TE values obtained from the WT (adjusted *p*-value of TE <0.05) (**Figure 2A**; **Supplementary Table S1**). Most of the transcripts that exhibited significant expression changes in the *tri* mutant were not affected in either the *urm* or *elo* mutants and may be responsible for the severe phenotypic changes caused by the lack of both wobbleU_34_ modifications.

We found more significant changes in ribosome occupancy (ribo-FClog_2_) than in transcriptional alterations (mRNA-FClog_2_) in all three wobbleU_34_-deficient mutant strains, indicating that the loss of either or both of the wobbleU_34_ modifications affected translation more than transcription (**Figure 2B**). The expression profiles of many more genes were altered in the *tri* mutant than in the *urm* or *elo* mutant (**Figure 2B**), indicating that the two distinct modifications in the wobbleU_34_ work together to properly translate many gene transcripts.

Transcripts showing significant expression changes with the log_2_ fold-change in TE (TE_FClog_2_) value >0 in the *tri* mutant accounted for two-thirds (755/1,123), which may have contributed to the large phenotypic changes observed in the *tri* mutants (**Figure 2C**). TE_FClog_2_ values >0 can be interpreted as ribosome occupancy on the transcript increasing during translation. However, this does not necessarily mean that their translation is accelerated; it may indicate ribosome pausing on these transcripts, leading to delayed or inefficient translation (Nedialkova and Leidel, 2015). The defects in the wobbleU_34_ modification might specifically affect the decoding of the cognate codons AAA, GAA, and CAA (referred to as [A/G/C]AA in this article) for Lys, Glu, and Gln, respectively. However, the frequency of [A/G/C]AA codon usage as well as the combined codon usage of each transcript did not correlate with their TE_FClog_2_ value in the *tri* mutant in any of the cases (**Supplementary Figure S3**).

Gene Ontology analysis showed that 145 of the 1,123 transcripts with significant TE_FClog_2_ values in the *tri* mutant were most remarkably enriched with the RNA-binding GO term (GO: 0003723) (**Figure 2D**, **Supplementary Tables S2 and S3**). Genes belonging to the RNA-binding category encode RNA-binding proteins (RBPs) involved in various RNA-related functions, such as ribosome biosynthesis and the posttranscriptional gene expression regulation (Glisovic et al., 2008; Bailey-Serres et al., 2009; Lee and Kang, 2016; Bazin et al., 2018; Chantarachot and Bailey-Serres, 2018; Dedow and Bailey-Serres, 2019; Muthusamy et al., 2021).

We compared expression levels of the 145 RBP transcripts between WT and the *tri* mutant (**Figure 2E**, left panel). The transcripts with mRNA-FClog_2_ values between −1 and 1 accounted for 88.3% (128/145) of the total. However, their ribo-FClog_2_ values ranged from −4.03 to 4.66, indicating that the differences observed in the TE_FClog_2_ values of these RBPs in the *tri* mutant could be primarily attributed to changes in ribosome occupancy. In addition, 82.1% (119) of the 145 RBP genes had TE_FClog_2_ values >0, suggesting that ribosome occupancy was substantially higher on those RBP transcripts in the *tri* mutant compared with the overall proportion of transcripts with TE_FClog_2_ values >0, which was 67.2% (755/1123) (**Figure 2E**, right panel). Genes that showed higher TE_FClog_2_ values >1 include several factors involved in rRNA biosynthesis, such as DEAD-box RNA helicase 1, *DRH1* (AT3G01540, also named *IRP6*), which is involved in 25S rRNA maturation (Palm et al., 2019); RNA helicase gene *ATRH7* (AT5G62190), which is also related to rRNA maturation (Huang et al., 2016); and the G-patch domain-containing protein gene *GDP1* (AT1G63980) (Kojima et al., 2018). Several rRNA-biosynthesis-related genes were also found among those with TE_FClog_2_ value <0 in the *tri* mutant, such as *IRP3* (AT4G17720), which is involved in pre-rRNA processing (Palm et al., 2019), and *GAF1* (RRP30, AT5G59980), which is a putative subunit of RNase P/MRP (**Figure 2E**) (Wang et al., 2012; Shaukat et al., 2021).

### 3.3 Deletion of both wobbleU_34_ modifications leads to accumulation of rRNA processing precursors

**Figure 3:**
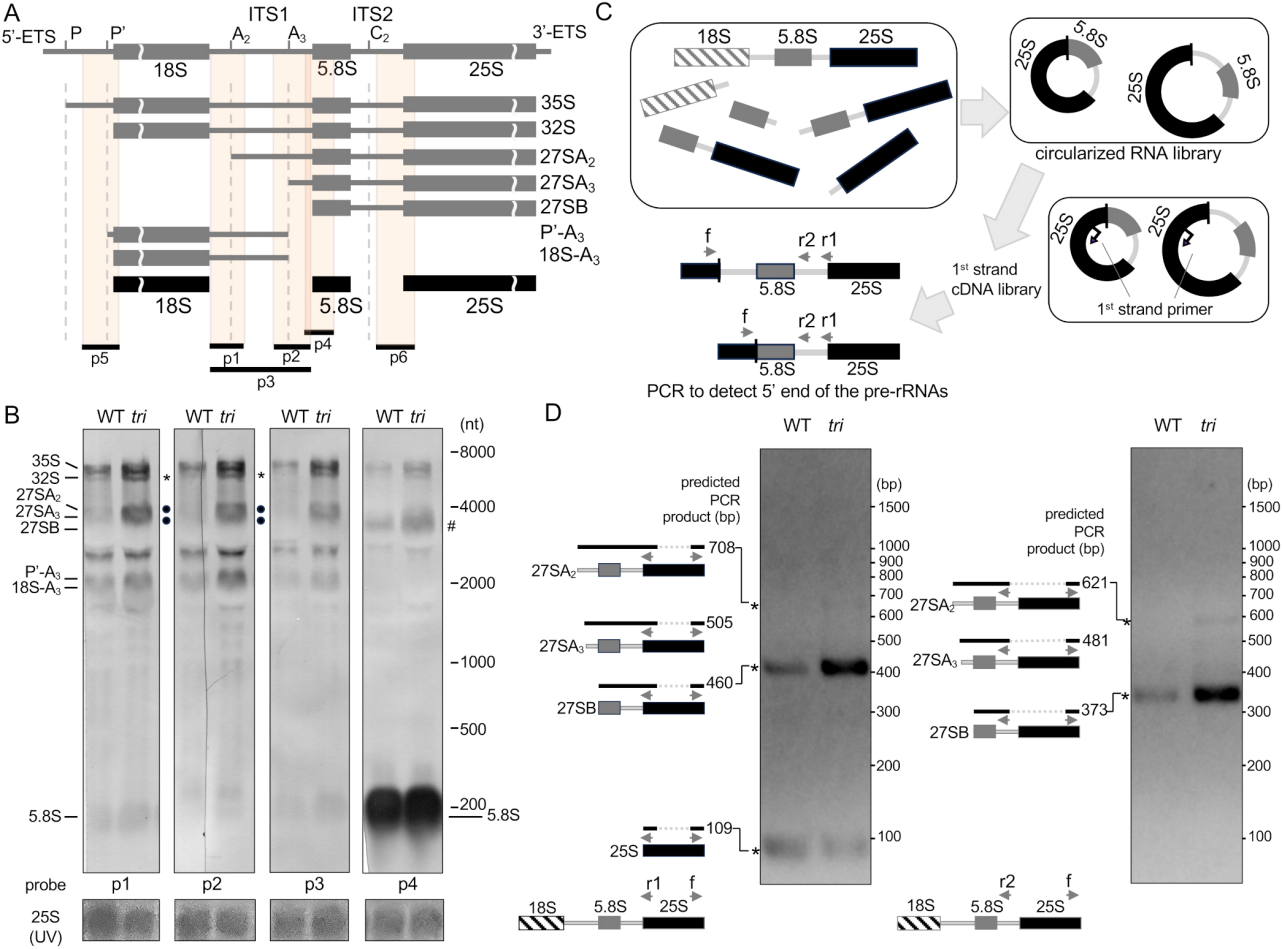
Accumulation of pre-rRNA intermediates in *tri* mutant lacking both wobbleU_34_ modifications. **(A)** Overview of rRNA maturation processes and pre-rRNA intermediates found in *Arabidopsis*. p1-p6 indicates the positions of the probes used in the Northern analysis. **(B)** Northern blot hybridization analysis of total RNA extracted from 11DAS seedlings of *Arabidopsis tri* and the WT. The positions of the final products and various intermediates are indicated. Abundant 28S rRNA bands detected under the UV light were shown as loading controls (the bottom panels). The abundant 5.8S rRNA bands in the right-most panel for the probe p4 serve as internal control. **(C)** Outline of method for detecting the 5′ end of pre-rRNA intermediates containing the 25S rRNA region using cRT-PCR. **(D)** Results of cRT-PCR analysis of *tri* mutant and WT. PCR-amplified fragments were separated via agarose gel electrophoresis. Deduced PCR fragments are schematically depicted at positions of expected sizes.

To explore the direct effects of the lack of both wobbleU_34_ modifications on rRNA biosynthesis, we performed northern blot analyses with total RNAs extracted from 11 DAS seedlings of the WT and the *tri* mutant to detect pre-rRNA precursors to be excised from the 35S pre-rRNA (**Figure 3A**). The results of northern blot analysis using probes p1, p2, and p3, which detect rRNA precursors retaining the internal transcribed spacer (ITS) 1 (ITS1) region, revealed that the *tri* mutant accumulated large amounts of 32S, 27SA_2_, 27SA_3_, P’-A_3_, and 18S-A_3_ pre-rRNA precursors (**Figure 3B**). This accumulation was not observed in the *urm* or *elo* mutants (**Supplementary Figure S4**). An additional rRNA processing intermediate, 27SB pre-rRNA, accumulated markedly in the *tri* mutant when using probe p4 (**Figure 3B**), whereas comparable levels of accumulation of the final product of 5.8S rRNA was detected in both the WT and the *tri* mutant samples. The accumulation of several distinct pre-rRNA processing intermediates indicated that rRNA maturation was significantly impaired in the *tri* mutant. rRNA can be biosynthesized through multiple pathways in *Arabidopsis* (Palm et al., 2019; Sáez-Vásquez and Delseny, 2019) (**Supplementary Figure S5**). The most common pathway involves the first cleavage within the ITS1 region of the 35S pre-rRNA precursor. Another rRNA biosynthesis pathway begins with cleavage of the 5′-external transcribed spacer (5′-ETS) region, which is similar to the rRNA biosynthesis pathway in yeast, and occurs in *Arabidopsis*. The 27SB pre-rRNA, consisting of the 5.8S-ITS2-25S region in both processes, is temporarily produced, cleaved within the ITS2 region, and produces mature 5.8S and 25S rRNAs. The accumulation of pre-rRNA processing intermediates in the *tri* mutant shown in **Figure 3B** indicates that the lack of both wobbleU_34_ modifications impairs rRNA maturation, especially the ITS2 cleavage step.

Next, we performed circular RT-PCR (cRT-PCR) analysis (**Figure 3C**) to precisely identify the rRNA intermediates accumulated in the *tri* mutant. With the single-stranded cDNA generated using a primer that bound near the 3′ end of the 25S sequence in the reverse direction, PCR was performed with another primer that bound near the 3′ end of 25S sequence (primer f) combined either with a primer (primer r1) that bound to the 5′ end of 25S or with a primer (primer r2) that bound to the ITS2 region using the obtained cDNAs as the template DNAs (**Figure 3D**). PCR amplified a 460-bp product with primers f and r1 corresponding to that derived from the 27SB pre-rRNA according to the results of DNA sequencing (**Figure 3D**, left). The 27SB pre-rRNA-derived PCR fragment was more strongly amplified using first-strand cDNAs prepared from the *tri* mutant RNAs than from the WT RNAs (**Figure 3D**, left). In addition, a small amount of the 708-bp PCR product containing the A_2_ site in the ITS1 region was detected only when using the cDNA from the *tri* mutant RNA, indicating 27SA_2_ pre-rRNA intermediate accumulation in the *tri* mutant. Furthermore, the predicted shorter fragments of the relevant sizes, 373 and 621 bp, were amplified with primers f and r2 (**Figure 3D**, right), further confirming the accumulation of 27SB and 27SA_2_ rRNA processing intermediates in the *tri* mutant.

### 3.4 Lack of both wobbleU_34_ modifications leads to ribosome pausing when the cognate [A/G/C]AA codon is positioned at the A site during translation of several rRNA maturation-related genes

**Figure 4:**
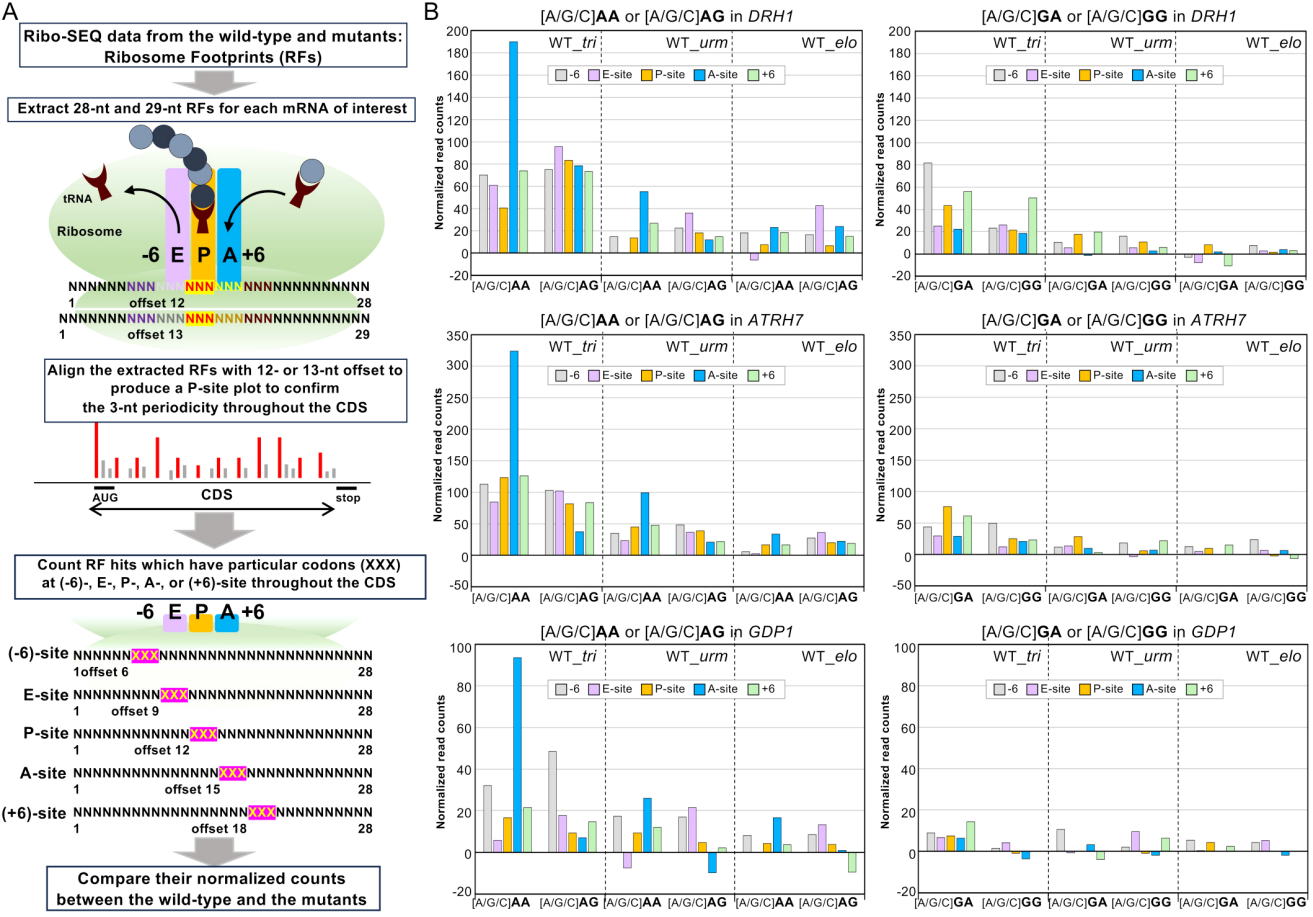
Ribosome footprints in relation to codons at different positions on translating ribosomes. **(A)** Methods to deduce the distinct site positions in ribosome footprints. **(B)** The ribosome footprint counts for the cognate [A/G/C]AA and [A/G/C]AG codons corresponding to the wobbleU_34_ containing tRNAs positioned at six different positions on translating ribosomes, including the P site and the A site, throughout the coding sequence of *DRH1* (top), *ATRH7* (middle), or *GDP1* (bottom) were compared between the WT and the *tri*, *urm*, or *elo* mutants, and their differences are plotted (left panels). As controls, the unrelated [A/G/C]GA and [A/G/C]GG codons were also analyzed and plotted (right panels). Values are shown as the difference in counts observed in the mutant relative to those observed in WT.

*DRH1*, *ATRH7*, and *GDP1* were included in the list of 145 RBP transcripts with significantly different TE_FClog_2_ values in *tri* compared with WT (**Figure 2E**) and were involved in rRNA biosynthesis (Palm et al., 2019; Huang et al., 2016; Kojima et al., 2018). To explore the direct effects of the wobbleU_34_ modifications on the translation of these transcripts, we first extracted the 100%-matched Ribo-seq records of 28- and 29-nt-long ribosome-protected mRNA fragments corresponding to the *DRH1* transcript. We generated a P site plot with 12 or 13 nt offset, respectively (**Figure 4A**). We thus confirmed the 3-nt periodicity starting from the ATG start codon to a codon just before the stop codon, which justified the P site assignments (**Supplementary Figure S6**). The number of ribosome-occupied reads containing [A/G/C]A**A** or [A/G/C]A**G** at the P site was then normalized to the total Ribo-seq read counts and compared between the WT and wobbleU_34_ modification mutants. Similarly, the ribosome-occupied reads containing [A/G/C]A**A** or [A/G/C]A**G**, either at their A or E site, as well as −6 or +6 nt relative to the P site, were separately counted and compared between the WT and mutants (**Figure 4A**). We found that the number of ribosome-occupied reads on the *DRH1* transcript was substantially higher especially when the [A/G/C]A**A** codon was positioned at the A site in the *tri* mutant (**Figure 4B**, top left). A similar trend was also observed for the transcripts of *ATRH7* and *GDP1* (**Figure 4B**, middle and bottom left panels). In contrast, this trend was not observed for the [A/G/C]**G**A codons unrelated to the wobbleU_34_ modification (**Figure 4B**, right panels).

## 4 Discussion

The simultaneous lack of both wobbleU_34_ modifications, mcm^5^ and s^2^, in *A. thaliana* was not lethal. However, the mutant plants lacking both wobbleU_34_ modifications, the *tri* mutant, exhibited severe developmental defects with severe growth retardation and morphological abnormalities in many tissues. These findings indicate that the complete absence of the two wobbleU_34_ modifications had a broadly adverse effect on cellular functions compared with single wobbleU_34_ modification-deficient mutants.

RNA-seq and Ribo-seq analyses indicated that the expression levels of many genes (>1,000) of the *tri* mutant significantly differed compared with those of the WT. Many of these genes were categorized as RBPs, being involved in various RNA-related processes such as ribosome biosynthesis and rRNA maturation. In addition to *DRH1*, *ATRH7*, and *GDP1* mentioned above, RNA helicase gene *RH5/STRS1*(AT1G31970) (Huang et al., 2016), one of the two *Arabidopsis* Nucleolin genes, *AtNUC-L2*(AT3G18610) (Pontvianne et al., 2007), another nucleolar-localized protein gene, *PESCADILLO* (AT5G14520) (Cho et al., 2013), and *IRP3/BPA1* (AT4G17720) were included in the list of 145 RBP transcripts. *IRP3* and *DRH1*(also named *IRP6*) genes were identified as *IRP* genes whose mutants exhibited pre-rRNA accumulation patterns distinct from that of the wild type (Palm et al., 2019). In the present study, among these 145 RBPs, we specifically selected *DRH1*, *ATRH7*, and *GDP1* for the detailed ribosome footprint analysis shown in **Figure 4** because of the following two criteria: i) relatively higher TE_FClog_2_l values (>1) and ii) a high enough number and coverage of Ribo-seq reads to be analyzed for ribosome profiling at single-base resolution throughout the coding sequences. *Arabidopsis drh1* mutant accumulated 27SA_2_ and 27SA_3_ pre-rRNA intermediates, suggesting that the processing of 27SB pre-rRNA at the C_2_ site was impaired in the *drh1* mutant (Palm et al., 2019; Sáez-Vásquez and Delseny, 2019). The accumulation of 27SA_2_ and 27SB pre-rRNA precursors was also detected in the *tri* mutant, which likely results from a delay in the cleavage of the ITS2 region in the pre-rRNA precursor (**Figure 3**). Ribosome footprint analysis indicated that, in the *tri* mutant, the ribosomes appeared to be pausing when the wobbleU_34_ modification-related cognate [A/G/C]AA codons were positioned at the A site in the translating ribosomes on the *DRH1* transcript (**Figure 4**). Thus, the observed increase in ribosome pausing during translation of the *DRH1* transcript probably caused delayed or insufficient supply of functional DRH1 protein, resulting in the accumulation of the pre-rRNA intermediates. A similar ribosome pausing was observed when the cognate [A/G/C]A**A** codons were positioned at the A site in the *tri* mutant for the *ATRH7* and *GDP1* transcripts (**Figure 4**). These results show that this trend in ribosome pausing at the A site of the cognate [A/G/C]A**A** codon is most likely a direct result of the deletion of both wobbleU_34_ modifications in the *tri* mutant. This biased ribosome pausing was more intense in the *tri* mutant than in the *urm* or *elo* mutants, indicating that the deletion of both wobbleU_34_ modifications synergistically had a negative effect on the entry of aminoacylated-tRNALys(UUU), -tRNALGlu(UUC), and -tRNAGln(UUG) into the A site of the translating ribosomes. Our observations in *Arabidopsis* are consistent with previous findings that ribosome pausing occurred when the [A/G/C]A**A** codons were positioned at the ribosome A site in similar complete wobbleU_34_ modification mutants of yeast and *Caenorhabditis elegans* (Nedialkova and Leidel, 2015).

Plant mutants in the biosynthesis of the translation apparatus, such as rRNA and ribosomal proteins (RPs), exhibit morphological and physiological abnormalities, often accompanied by an impaired auxin response (Bailey-Serres et al., 2009; Zhou et al., 2010; Bazin et al., 2018; Sáez-Vásquez and Delseny, 2019). The phenotypic defects observed in the *tri* mutant (**Figure 1**) are similar to those observed in mutant RP genes (Nishimura et al., 2005; Horiguchi et al., 2011). RPs account for a significant portion of RBPs in *Arabidopsis* (Bailey-Serres et al., 2009). However, only six RP genes were included in the list of 1,123 transcripts that showed significantly altered translation efficiencies due to the lack of wobbleU_34_ modifications (**Supplementary Figure S7**). This is presumably because RP gene transcripts are relatively highly expressed but preferentially use AAG codon for Lys instead of AAA (**Supplementary Figure S8**) and that the presence or absence of wobbleU_34_ modifications in the corresponding tRNA does not affect the decoding efficiency of AAG codon, as shown in the present study (**Figure 4B**) and the literature (Nedialkova and Leidel, 2015).

As mentioned above, similar to the *tri* mutant, the *drh1* mutant accumulated pre-rRNA processing intermediates, and this accumulation was more pronounced after auxin treatment, which enhances pre-rRNA synthesis (Palm et al., 2019). This suggests a rate-limiting role of DRH1 in pre-rRNA processing under such conditions. In addition, AtRH7 is involved in rRNA processing, and the *ATRH7* gene-knocked mutant exhibits similar phenotypes as the auxin-related mutants (Huang et al., 2016). These findings suggest a possible link between auxin response and rRNA biosynthesis. From this perspective, in the *tri* mutant, the lack of both wobbleU_34_ modifications causes ribosome pausing during translation of proteins involved in rRNA maturation, including DRH1 and/or ATRH7, which delay rRNA biosynthesis and may ultimately affect the auxin response, resulting in morphological abnormalities in various organs and tissues.

Interestingly, some organelle-related gene transcripts, such as *PPR2* (AT3G06430), which interact with chloroplast 23S rRNA (Lu et al., 2011), and *RNR1* (AT5G02250), which is involved in both mitochondrial and chloroplast rRNA maturation (Bollenbach et al., 2005), are included in the list of the 145 RBP transcripts. Although their TE_FClog_2_ values were lower than those of *DRH1*, *ATRH7*, and *GDP1*, organellar rRNA biosynthesis may also be affected in the *tri* mutant.

In addition to these rRNA biosynthesis-related gene transcripts, splicing factor *PRP4*, which is involved in seed development (Raab and Hoth, 2007), *PPR4* (AT5G04810), which modulates the trans-splicing of *rps12* transcripts in chloroplasts (Lee et al., 2019), and *HOG1* (AT4G13940), which is required for DNA methylation-dependent gene silencing (Rocha et al., 2005), are also included in the 145 RBP list, suggesting that other RNA-related functions such as mRNA-splicing or RNA silencing might also be affected in the *tri* mutant. Further work will be needed to clarify this point.

A recent report by Linder et al (Linder et al., 2025). proposed that interactions between m_6_A-modified codons in mRNAs and wobbleU_34_ modifications in tRNAs may affect mRNA decay in cultured human tumor cells. It remains unclear whether this interaction also affects mRNA decay in plants, such as *Arabidopsis*, and this issue awaits future studies.

It should be noted that there are interesting differences in the observed phenotypes between mice/flies and *Arabidopsis* caused by the lack of two wobbleU_34_ modifications. Regarding cognate codon usage, mice and flies exhibit a relatively higher usage of XAG codons over XAA codons, whereas *Arabidopsis* shows nearly equal usage of these codons (**Supplementary Figure S9**). Although it is unclear whether these differences in codon usage are correlated with differences in the lethality of the mutants, animals and plants exhibit many differences during development and growth. Considering that the loss of tRNA wobbleU_34_ modifications in plants, although not lethal, causes severe growth retardation and morphological abnormalities, such effects, if they occur during the development and growth of animals, might be more critical for their survival.

Our findings highlight the physiological importance of the wobbleU_34_ modification in plants and further provide a clue to elucidate a close link between translational capacity and the development of tissues and organs in multicellular organisms.

## Data Availability

The original contributions presented in the study are included in the article/**Supplementary Material**, further inquiries can be directed to the corresponding author.
