# Supplementary material for: Loss of both wobbleU34 modifications in mcm5s2U tRNAs impairs rRNA biosynthesis, growth, and development in Arabidopsis thaliana
Source: Front Plant Sci. 2025 Dec 3;16:1681927. doi: 10.3389/fpls.2025.1681927 (PMC12708939; doi:10.3389/fpls.2025.1681927)
Supplement: Supplementary file 1 [file DataSheet1.pdf]

*Supplementary Material*

**Loss of both wobbleU$_{34}$ modifications in mcm$^5$s$^2$U tRNAs impairs rRNA biosynthesis, growth, and development in *Arabidopsis thaliana***

**Yumi Nakai[1*], Yukio Kurihara[2], Yuko Makita[2,3], Gorou Horiguchi[4,5], Kosei Iwabuchi[6], Akiko Harada[6], Masato Nakai[7], Takato Yano[1]**

*** Correspondence:**
Yumi Nakai
yumi.nakai@ompu.ac.jp

# 1 Supplementary Figures and Tables

## 1.1 Supplementary Figures

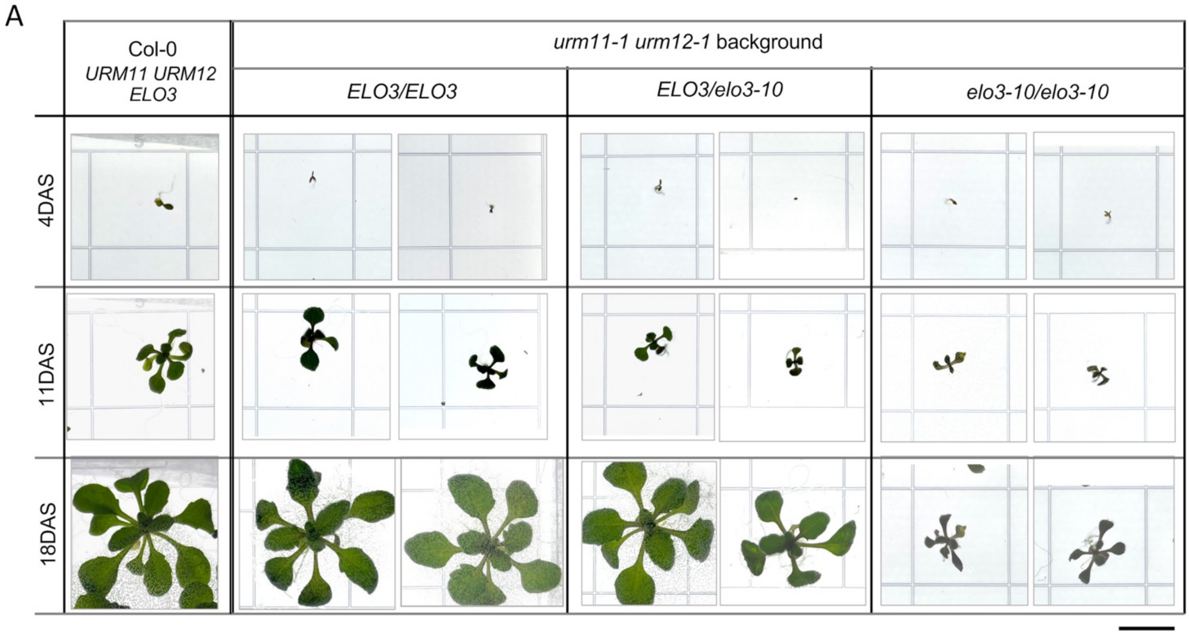


B

| | *urm11-1 urm12-1* background | | |
|---|---|---|---|
| | *ELO3*/*ELO3* | *ELO3*/ *elo3-10* | *elo3-10*/*elo3-10* |
| Proportion (%) (s.d.) | 26.3 (4.8) | 50.9 (3.5) | 13.6 (2.9) |

**Supplementary Figure S1.** Segregation analysis of progenies derived from the parental mutant line carrying double homozygous *urm11-1 urm12-1* and heterozygous *ELO3*/*elo3-10* mutations. Seeds were analyzed in three biological triplicates, using 72-98 seeds per replicate. **(A)** Growth of the seedlings of *ELO3/ELO3*, *ELO3*/*elo3-10*, and *elo3-10*/*elo3-10* under the homozygous *urm11-1 urm12-1* background was compared at 4, 11, 18 DAS together with that of the WT. Representative images are shown. Scale bar, 1 cm. **(B)** Segregation of genotypes in progenies is shown in proportions. The mean value with standard deviation (s.d.) for each genotype is shown.

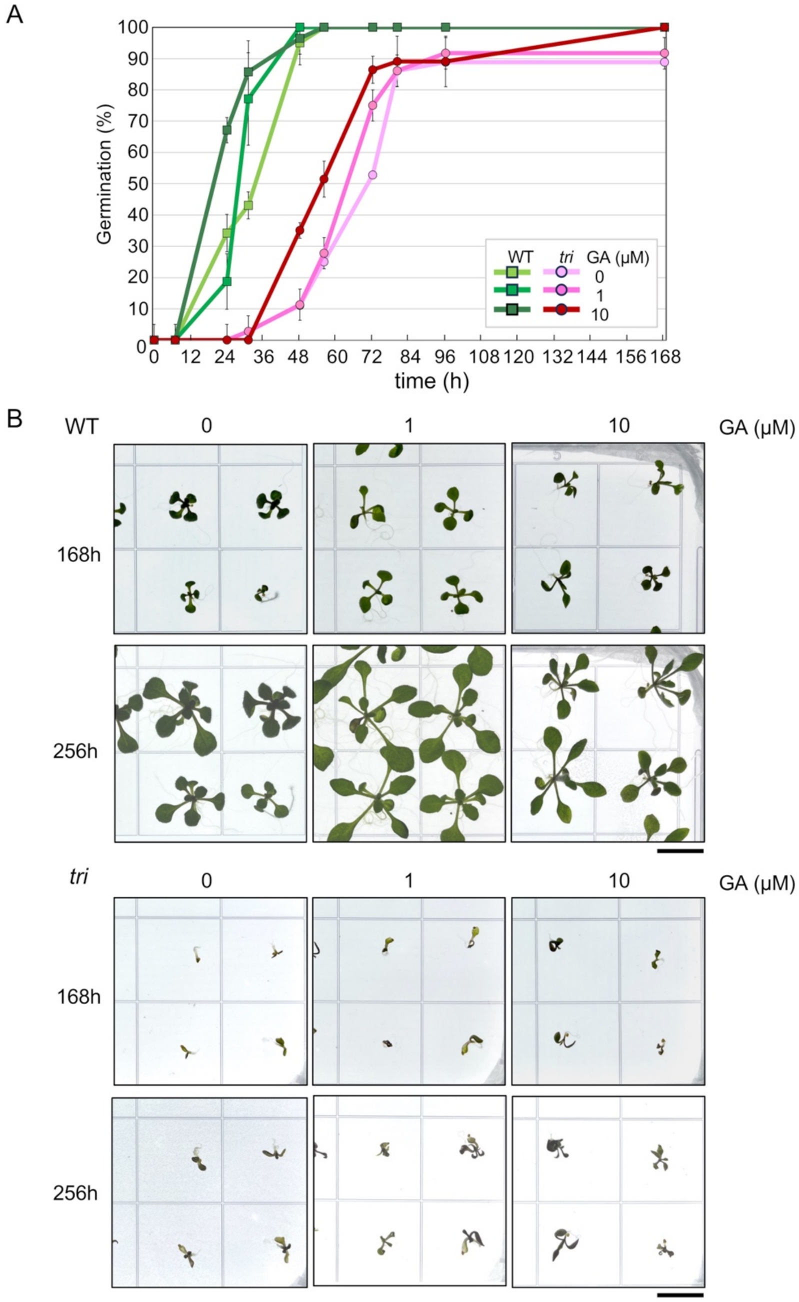


**Supplementary Figure S2. (A)** Germination analysis of the WT and the *tri* mutant seeds. After being sown on half-strength MS plates containing increasing concentrations of gibberellin (GA), followed by stratification, seeds were transferred to a growth chamber at 22°C (this time point was set as 0 hour) and were grown under normal growth conditions. Time of germination was defined when both the radicle and the cotyledons emerged through the seed coat. Each germination ratio (%) is indicated as the average of biological duplicates with error bars. **(B)** WT and the *tri* seedlings grown with or without GA in the medium, as in **(A),** were compared after 168 and 256 h of growth. Scale bar, 1 cm.

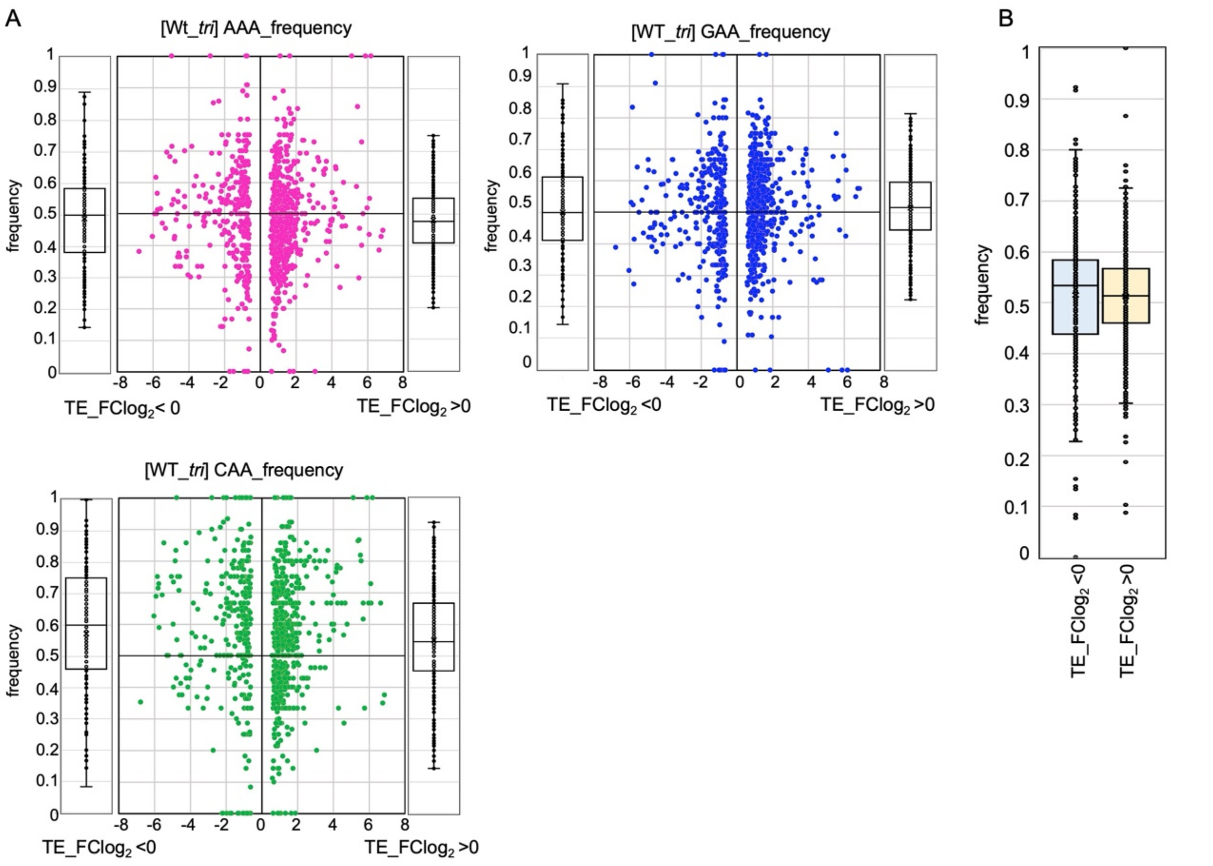


**Supplementary Figure S3. (A)** Frequency of occurrence of codons [A/G/C]AA (AAA for Lys, GAA for Glu, CAA for Gln) with wobble$U_{34}$ modification in transcripts with differential expression between the WT and *tri* mutant. The frequency of occurrence of target codons in each transcript was calculated using the occurrence of [A/G/C]AA codons against the total number of [A/G/C]AA and [A/G/C]AG codons in each transcript, plotted against TE_FC$\log_2$. Box plots show the frequency distribution of each [A/G/C]AA codon in the entire set of transcripts with negative or positive TE_FC$\log_2$ values. Bars within box plots indicate mean values. **(B)** Box plots showing average combined frequency of three [A/G/C]AA codon occurrences in transcripts differentially expressed between the WT and *tri*, with TE$\log_2$ values divided into positive and negative values.

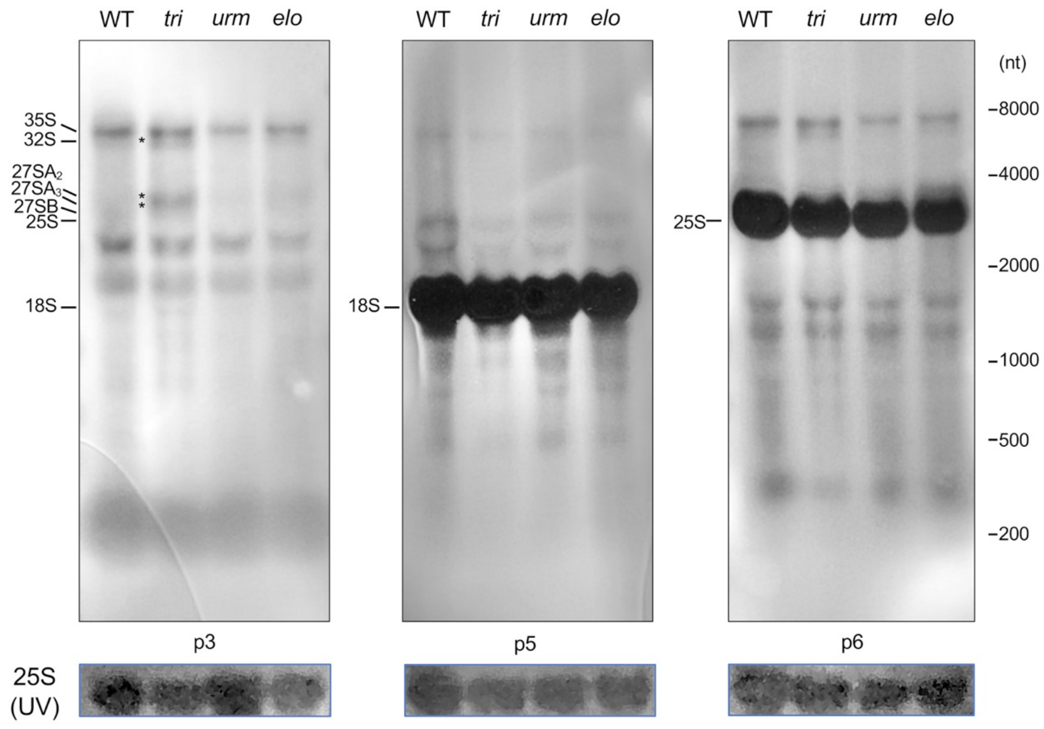


**Supplementary Figure S4.** Northern blotting of total RNA extracted from 11 DAS seedlings of *Arabidopsis* wobbleU$_{34}$-modification-deficient mutants (*tri*, *urm*, and *elo* mutants) and WT, obtained using digoxigenin-labeled DNA probes p3, p5, and p6. The positions of the probes are shown in Figure 3*A*. Positions of various intermediates as well as mature 18S rRNA and 25S rRNA are indicated. Pre-rRNA precursors described in the text are marked with asterisks. As a loading control, UV-visualized 25S rRNA is shown at the bottom.

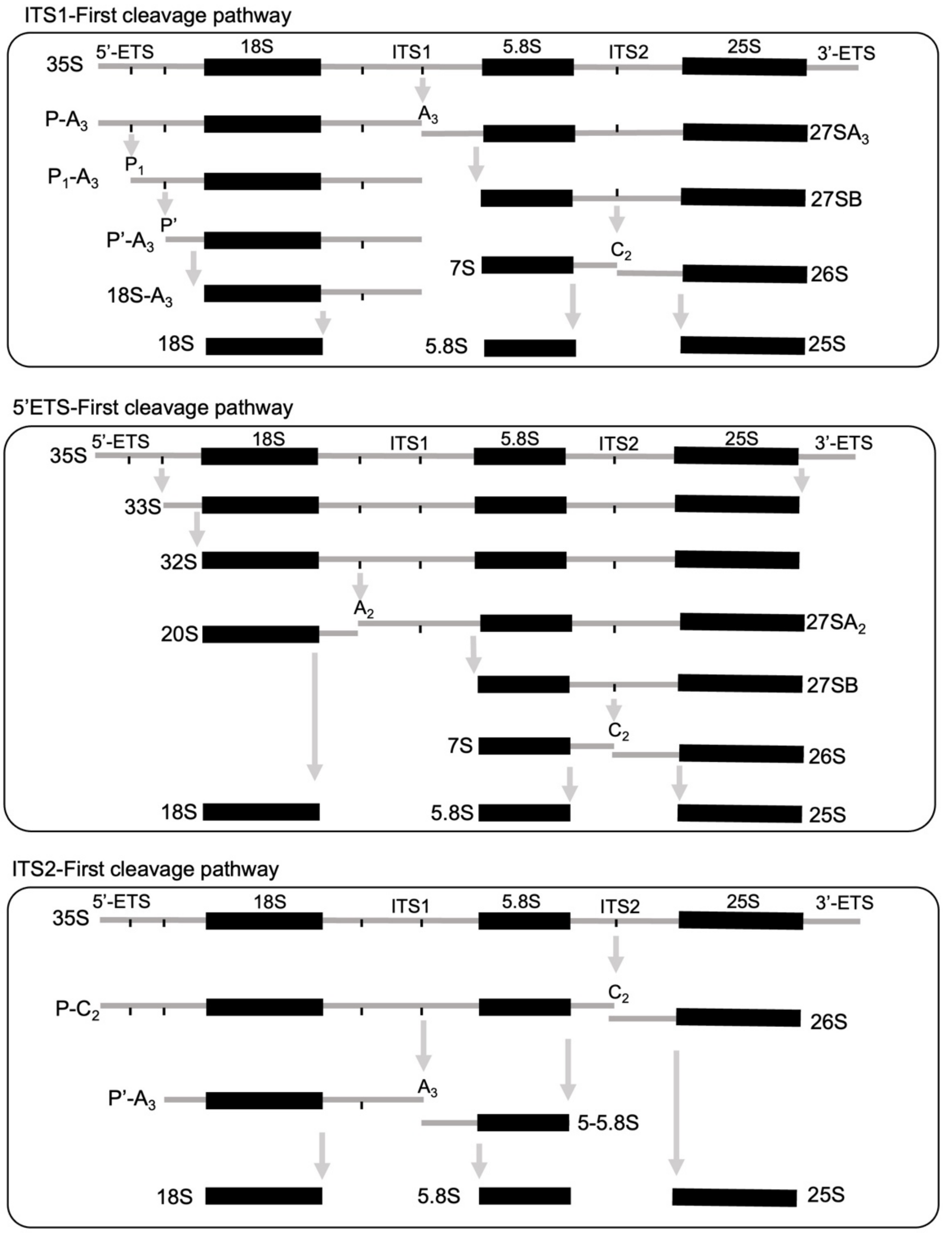


**Supplementary Figure S5.** *Arabidopsis* rRNA biosynthesis pathways: ITS1-first, 5'-ETS-first, and plant-specific cleavage pathways.

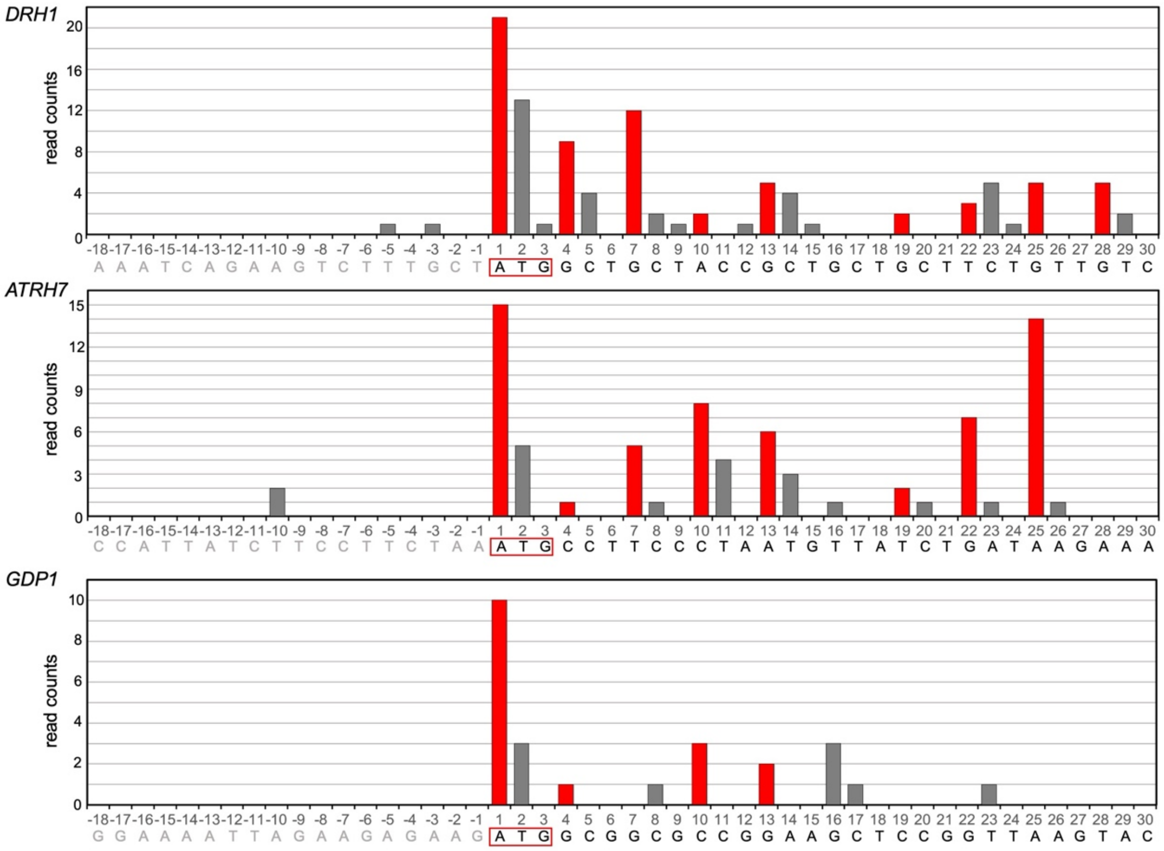


**Supplementary Figure S6.** P site plots for transcripts of *DRH1* (top), *ATRH7* (middle), and *GDP1* (bottom). Ribosome footprints of 28 and 29 nt were aligned throughout their transcripts with 12- or 13-nt offsets, respectively, to deduce their P site locations. Ribo-seq hit counts (y-axis) are plotted versus positions of the first nucleotide of the deduced P site codon (x-axis). The first A of the initiator ATG codon is defined as 1. Red bars indicate continuous 3 nt periodicity starting from the initiator ATG, and gray bars indicate other read counts. Values are shown for 18 nucleotides upstream and the first 30 nucleotides of the coding sequence.

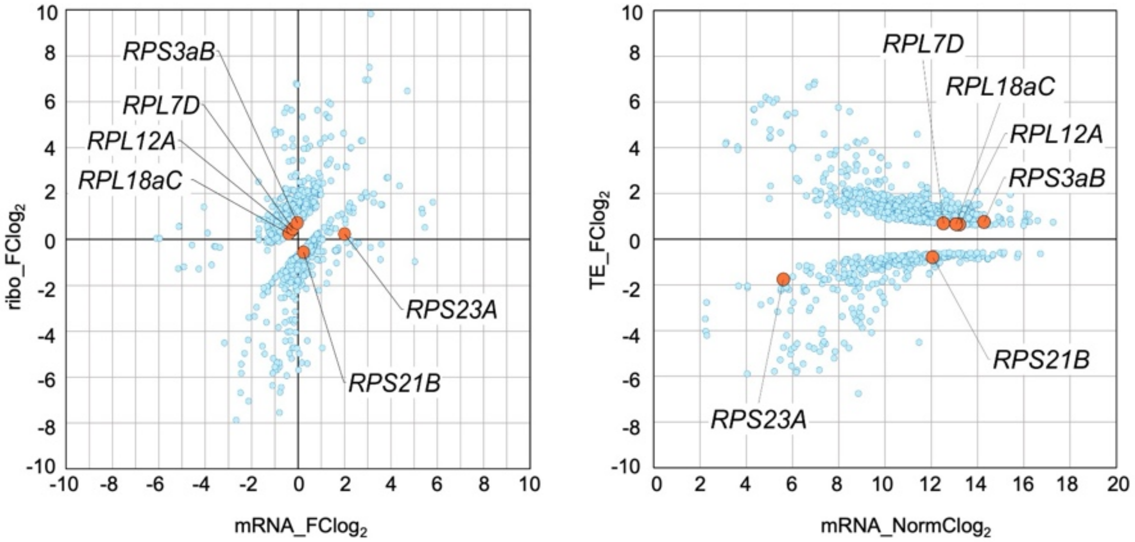


**Supplementary Figure S7.** Among the 1123 transcripts (light blue circles) that showed significant changes in translation efficiency (TE) in the *tri* mutant compared to the WT, six transcripts categorized as ribosomal proteins (RPs) by GO analysis are indicated by red circles. The $\log_2$-fold change in ribosome occupancy (ribo_FClog$_2$) was plotted against the $\log_2$-fold change in mRNA expression (mRNA-FClog$_2$) (left), and the $\log_2$-fold change in translation efficiency (TE_FClog$_2$) was plotted against the $\log_2$ value of the normalized mRNA read count (right).

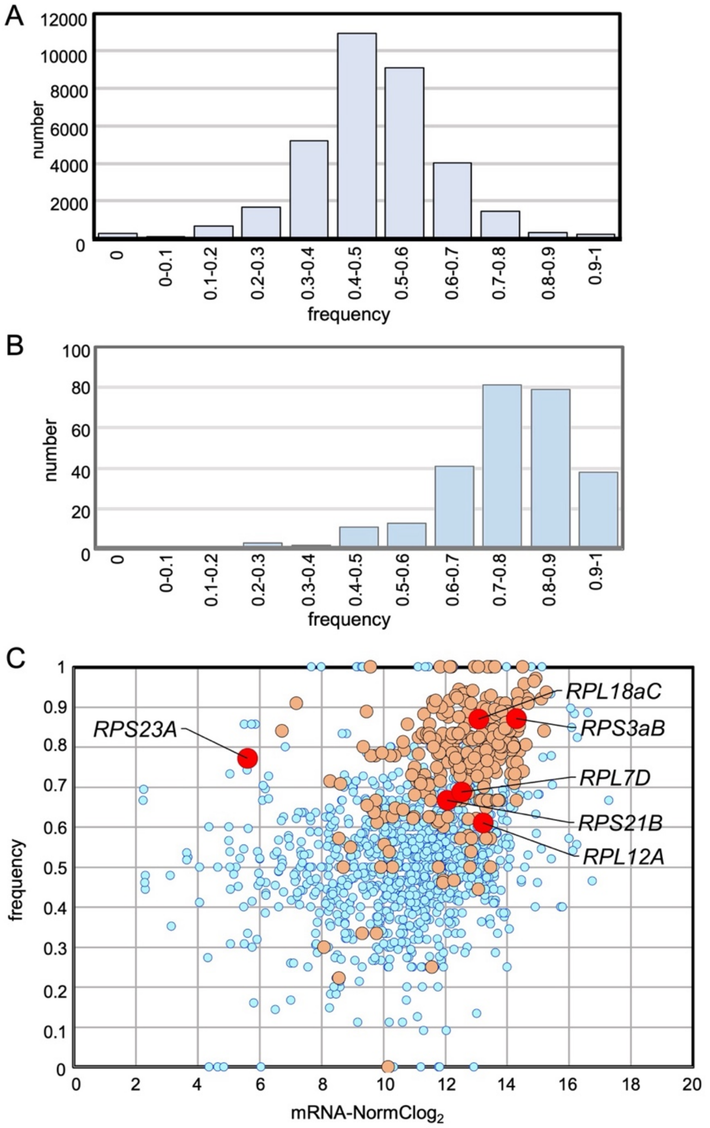


**Supplementary Figure S8.** Frequency of occurrence of Lys (AAG) codon against the total number of Lys (AA[A/G]) codons in 33899 transcript genes **(A)** and cytosolic RP genes **(B)** in *Arabidopsis thaliana*. Numbers of gene transcripts are plotted (y-axis). **(C)** The frequency of the AAG codon was plotted against the mRNA-NormClog$_2$ of each transcript. Values of the 1123 transcripts that exhibited significant TE_FClog$_2$ values between the WT and the *tri* mutant are indicated by light blue circles, among which six RP gene transcripts are highlighted by red circles. Values of the RP gene transcripts not included in the list of 1123 transcripts are indicated by orange circles.

| | Sc | Ce | Hs | Mm | At | Nt |
|---|---|---|---|---|---|---|
| AAA | 0.65 | 0.68 | 0.37 | 0.30 | 0.47 | 0.49 |
| AAG | 0.35 | 0.32 | 0.63 | 0.70 | 0.53 | 0.51 |
| GAA | 0.85 | 0.73 | 0.35 | 0.32 | 0.53 | 0.60 |
| GAG | 0.15 | 0.27 | 0.65 | 0.68 | 0.47 | 0.40 |
| CAA | 0.84 | 0.78 | 0.11 | 0.11 | 0.62 | 0.66 |
| CAG | 0.16 | 0.22 | 0.89 | 0.89 | 0.38 | 0.34 |

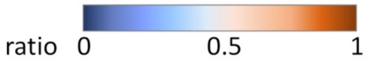


**Supplementary Figure S9.** Comparison of synonymous codon usage for lysine (AAA or AAG), glutamic acid (GAA or GAG), and glutamine (CAA or CAG) of various organisms: Sc, *Saccharomyces cerevisiae*; Ce, *Caenorhabditis elegans*; Hs, *Homo sapiens*; Mm, *Mus musculus*; At, *Arabidopsis thaliana*; Nt, *Nicotiana tabacum*. Values are shown as ratios in the heatmap visualization.

## 1.2 Supplementary Tables

**Supplementary Table S1.** Differentially expressed gene analysis between the wobbleU$_{34}$ modification mutants and the WT is summarized in Figure 2A-2C. A transcript identifier with an underscore and a number denotes the presence of different coding regions in the transcript.

**Supplementary Table S2.** GO terms of genes for transcripts with significantly different translation efficiencies in the *tri* mutant compared with those of WT. The values used for the GO-plot shown in Figure 2D are shown.

**Supplementary Table S3.** Genes for 145 transcripts belonging to the RNA-binding protein (RBP) category were identified via Gene Ontology analysis of 1123 transcripts that exhibited significantly different translation efficiencies in the *tri* mutant compared with WT. The list is sorted by TE_FClog$_2$ values. A transcript identifier with an underscore and a number denotes the presence of different coding regions in the transcript.

**Supplementary Table S4.** Oligonucleotides used in this study.
